# Mr. Hutchinson's Case of Lacerated Femur

**Published:** 1801-08

**Authors:** Benjamin Hutchinson

**Affiliations:** Member of the Royal College of Surgeons. Southwell, Notts.


### *To the Editors of the Medical and Physical Journal.*

GENTLEMEN,

SHOULD the following ſingular caſe, which has lately fallen under my care, appear to you ſufficiently intereſting to merit a page in your valuable Journal, it is much at your ſervice.

*Southwell, Notts.*
*July* 3, 1801.

I am, &c.
BENJAMIN HUTCHINSON,
Member of the Royal College of Surgeons.

---

ON the 30th of laſt May, I was called to Thomas Jephſon, twelve years of age, and ſervant to Mr. Chadwin, a farmer at Winkburn, in this county. It was ſuppoſed that he muſt be dead from an exceſs of hæmorrhage before I could poſſibly reach the village, at a diſtance of three miles and a half. On my arrival, however, I found the boy in a ſtate of ſyncope; and on enquiring into the particulars of the accident, was informed, that on returning from ploughing, he rode a reſtive horſe, and carried ſome plough irons before him; the horſe beginning to be unmanageable, the boy threw the implements from him, one of which ſtuck perpendicularly in the ground: he was immediately thrown, and fell with the poſterior part of his right thigh upon this iron, which tore its way through the

---

the anterior part, dragging with it in its courſe the femoral artery, juſt below its bifurcation into the profunda. The artery was completely divided, and its lacerated extremities were forced externally through the wound. Owing, I conſider, partly to the contraction of the veſſel, to the coagulum formed within its tube, and probably more particularly to the complete ſtate of ſyncope which ſupervened, the hæmorrhage had ceaſed before I had reached the village. This large artery being ſo immediately in view, I had no difficulty in ſecuring with tape ligatures, each divided extremity; and from the evident and impetuous pulſation, convinced that my patient was living, I introduced the veſſel within the gaping laceration of muſcles, leaving the ends of the ligatures externally. By means of ſutures and ſlips of adheſive plaſter, the lips of the wound were ſatisfactorily approximated, and having applied ſimple dreſſings, I ſecured them by a long flannel roller. The deliquium was removed by the irritation occaſioned by the ligatures and ſutures.

Conſidering my patient in the ſame ſtate as after the operation for popliteal aneuriſm, I left him with an impreſſion of anxiety on my mind, not without hopes, however, that the profunda, with the anaſtomoſing arterial ramifications, would be ſufficient to furniſh a due circulation for the nouriſhment of the limb. On the following day, I found he had paſſed a reſtleſs night, notwithſtanding he had taken a draught with twenty-five drops of tincture of opium; the wound was painful; he was yet free from fever, and the limb retained its natural heat. The quantity of opiate having been increaſed the ſecond night to thirty drops, he was not ſo reſtleſs, ſtill, however, ſuffering conſiderable uneaſineſs, and complaining of a numbneſs in the whole limb. Care was taken to keep his bowels in a ſufficiently open ſtate; and with the aſſiſtance of the ſame quantity of opium, he again paſſed a comfortable night. The degree of pain in the wound varied until the ſixth day, when I cautiouſly removed the dreſſings, which were completely moiſtened by a copious effuſion of coagulable lymph from the mouths of the divided lymphatics. On the day following my patient was ſeen by Mr. Beechor, a moſt reſpectable ſurgeon of this place, and Mr. Thompſon, a well informed practitioner of Newark, who concurred in opinion that the boy was doing better even than might have been excepted. Appearances now continued much in our favor; the pain was inconſiderable, and the extremity gradually recovered its regular temperature and ſenſation. Nothing occurred to interrupt the healing of the wound. On the twelfth day from the accident, the ligatures were detached from the artery, and in the courſe of five weeks the wound was comcompletely healed, and the boy recovered the perfect ufe of his limb.

